# Uterine manipulator requirement in laparoscopic surgery of Ectopic Pregnancy

**DOI:** 10.12669/pjms.36.2.1294

**Published:** 2020

**Authors:** Emrah Beyan, Ahkam Goksel Kanmaz, Adnan Budak, Volkan Emirdar, Sadettin Oguzhan Tutar, Abdurrahman Hamdi Inan

**Affiliations:** 1 Dr. Emrah Beyan, MD. Department of Obstetrics and Gynecology, Tepecik Education and Research Hospital, Izmir, Turkey; 2 Dr. Ahkam Goksel Kanmaz, MD. Department of Obstetrics and Gynecology, Tepecik Education and Research Hospital, Izmir, Turkey; 3 Dr. Adnan Budak, MD. Department of Obstetrics and Gynecology, Tepecik Education and Research Hospital, Izmir, Turkey; 4 Dr. Volkan Emirdar, MD. Department of Obstetrics and Gynecology, Medical Park Hospital, Izmir, Turkey; 5 Dr. Sadettin Oguzhan Tutar, MD. Department of Obstetrics and Gynecology, Ardahan State Hospital, Ardahan, Turkey; 6 Dr. Abdurrahman Hamdi Inan, MD. Department of Obstetrics and Gynecology, Bornova Turkan Ozilhan State Hospital, Izmir, Turkey

**Keywords:** Complication, Ectopic pregnancy, Laparoscopy, Uterine manipulator

## Abstract

**Objective:**

The advantages of laparoscopic surgery used in the treatment of ectopic pregnancy is well-known; however, the efficacy of uterine manipulators remains unknown. In this study, we aimed to investigate the efficacy of uterine manipulators in the laparoscopic treatment of ectopic pregnancy.

**Methods:**

Overall, 118 patients who underwent laparoscopy due to ectopic pregnancy in Department of Obstetrics and Gynaecology at Tepecik Education and Research Hospital between January 2010 and January 2018 and who met the inclusion criteria were included in the study. Groups of patients undergoing surgery with or without the use of a uterine manipulator were compared in terms of demographic data, operative and postoperative results.

**Results:**

No difference was noted between the groups in terms of age, parity, body mass index, smoking, side of ectopic pregnancy mass, previous operations and pregnancy type. However, the size of ectopic pregnancy mass measured by ultrasonography was significantly larger (p = 0.006) and the operation time was significantly shorter (p<0.001) in the group where uterine manipulators were not used than in the uterine manipulator group.

**Conclusion:**

We concluded that not using a uterine manipulator in laparoscopic procedures for ectopic pregnancy did not increase operative complications and that operation time was higher in procedures using uterine manipulators.

## INTRODUCTION

Whether planned or unplanned, approximately 2% of all pregnancies occur outside the uterus, and a long follow-up and treatment process is anticipated in patients diagnosed with ectopic pregnancy. However, early diagnosis and appropriate treatment of ectopic pregnancy, which is the most common cause of maternal deaths in early pregnancy, significantly reduces mortality and morbidity; moreover, fertility can be often maintained in such patients.^1^

Stovall et al. reported a safe outpatient treatment protocol in patients with ectopic pregnancy on the use of methotrexate for ectopic pregnancy that was first described in 1982.^2^ Approximately 90% of ectopic pregnancies have been successfully treated with methotrexate, which has been used since nearly 35 years, without the need of any surgical intervention.^3^

Patients with contraindications to methotrexate require surgical intervention, and laparoscopy has considerable advantages compared with laparotomy.^4^ However, laparoscopic surgery has its own complications, such as vascular, intestinal, nerve or bladder injuries during entry into the abdominal cavity, infection in the entry areas and subcutaneous emphysema due to pneumoperitoneum, pneumothorax, cardiac arrhythmia and carbon dioxide retention.^5^

Some surgeons use uterine manipulators during operative or diagnostic laparoscopy in patients with a uterus to facilitate manipulation, and the use of uterine manipulators has its complications as well. Although it is thought to provide convenience during surgery, complications such as uterine perforation, uterine vascular, bowel or bladder injuries and vaginal lacerations associated with the use of uterine manipulator and problems such as retention of a part of the uterine manipulator in the vagina can be encountered.^6^

In this study, we aimed to demonstrate whether the use of uterine manipulators during laparoscopic salpingectomy or salpingostomy is necessary in the surgical treatment of ectopic pregnancy. To our knowledge, this is the first study to investigate uterine manipulator requirement in laparoscopic surgery for ectopic pregnancy.

## METHODS

This is a retrospective-cohort study about uterine manipulator necesssity for laparoscopic treatment of ectopic pregnancy. Patients who were hospitalized with a diagnosis of ectopic pregnancy in the Department of Obstetrics and Gynaecology at Tepecik Education and Research Hospital between January 2010 and January 2018 and who were scheduled to undergo laparoscopic procedures were reviewed using the hospital information system after approval was obtained from Ministry of Health, Tepecik SUAM Ethics Committee (Reference number: 20187/10-8). A total of 118 patients who were diagnosed with ectopic pregnancy and those with contraindications to methotrexate, who were scheduled to undergo laparoscopic treatment for not consenting to methotrexate therapy or due to previous unsuccessful methotrexate therapy, who had not undergone additional surgical interventions such as tubal ligation or dilatation and curettage during surgery and who did or did not undergo surgery using perioperative uterine manipulation were included in the study. Only patients with ectopic pregnancy located in the fimbria, infundibulum and ampulla were included in this study, whereas those with caesarean scar, heterotopic, cornual ectopic, ovarian ectopic and abdominal pregnancies were excluded

Demographic data such as age, parity, body mass index (BMI), smoking, chronic systemic diseases, preoperative serum BhCG and previous abdominal surgery were recorded for 61 patients who had undergone laparoscopy without uterine manipulators for ectopic pregnancy and 57 patients who had undergone laparoscopy with uterine manipulators. Operative data such as operation time, estimated blood loss (ml), uterine manipulator use and operative complications were recorded from the operation notes. The operation time was defined as the time between opening and closing the skin incision, return of bowel function was defined as the first gas passage after extubating the patient, cervical laceration was defined as all cervical lacerations that required intervention during the insertion or removal of uterine manipulators and subcutaneous emphysema was defined as the diffusion of insufflating carbon dioxide during operation within subcutaneous tissues.

In our clinic choice of surgery for ectopic pregnancy depends on higher levels 5000 u/mL of BhCG and detecting above 4 cm ectopic mass or peritoneal fluid or fetal cardiac activity or rupture ectopic mass in ultrasound. The use of uterine manipulator for laparoscopic surgery of ectopic pregnancy in our clinic depends on surgeons preference. Patients operated with uterine manipulators were operated in lithotomy position, whereas those who were not operated with uterine manipulators were operated in the supine position. At our clinic, patients who are scheduled to undergo manipulator placement in laparoscopic procedures are evaluated with bimanual vaginal examination after appropriate perineal and vaginal cleansing in the lithotomy position. Following speculum insertion, the cervix is held with the tenaculum, after placing a traction on the uterus, uterine cavity is entered with a hysterometry and the uterine cervix is dilated with 6–7 mm hegar dilators equal to the width of the uterine manipulator tip.

The uterine manipulator is advanced through the cervical canal, and when it reaches the cavity, the manipulator tip balloon is inflated with a 2–3 ml sterile saline to fix the manipulator. All these procedures are monitored by a laparoscope, and if uterine perforation is observed, primary repair is laparoscopically performed. All patients included in the study had laparoscope insertion from the umbilicus with a 10-mm trocar and whole abdomen exploration, followed by a 5-mm trocar insertion 2–3 cm medial to the left anterior superior iliac spine and another 5-mm trocar insertion between this port and the umbilicus. In the presence of intraabdominal haemorrhagic fluids, the patients who were included in the study received partial salpingectomy with a bipolar tissue sealer or salpingostomy with a monopolar laparoscopic needle-tipped electrode following the aspiration, based on the clinical condition of the patient or the surgeon’s preference.

### Statistical analysis

The results were presented as frequency and percentage. Normality tests were selected in accordance with the number of ectopic pregnancies, and a normal distribution pattern was accepted if p > 0.05. The results were presented as mean ± standard deviation (SD) for normally distributed data and median (range) for non-normally distributed data. The chi square test or Fisher’s exact test was used for intergroup differences of categorical variables based on the number of data. For univariate analyses, one sample t-test was used for parametric variables, and the Mann-Whitney U test was used for non-parametric variables. p <0.05 was considered statistically significant. Statistical analyses were performed using SPSS 22.0 for Windows (SPSS Inc., Chicago, IL).

## RESULTS

No statistically significant difference was noted between the groups in terms of demographic data. The mean age, BMI and preoperative BhCG values were higher in the uterine manipulator group than in the group where uterine manipulators were not used, but the difference was not statistically significant (P = 0.450, P = 0.265, and P = 0.968, respectively). The size of the ectopic pregnancy mass measured by preoperative ultrasonography was 27.4±4.2 mm in the uterine manipulator group and 30.4±5.9 mm in the group where uterine manipulators were not used, and the difference was statistically significant (P = 0.006). No statistically significant difference was found between the groups in terms of previous abdominal surgery and type of pregnancy (spontaneous or assisted reproductive techniques). The clinical data of patients are summarised in Table-I.

**Table-I:** Patient clinical data.

	Uterine manipulator used n = 61 (number(%), range or mean±SD)	Uterine manipulator not used n = 57 (number(%), range or mean±SD)	p
Age	30.6 ± 5.3	29.9 ± 5.5	0.45
Parity	2 (0–5)	2 (0–4)	0.428
BMI g/m2)	27.4 ± 4.2	26.6 ± 3.5	0.265
Smoking	19 (31.5)	15 (26.5)	0.354
Preoperative BhCG (mIU/ml)	3400 (1640-12100)	3100 (1950-9500)	0.968
Size of ectopic mass with USG (mm)	27.4 ± 5.7	30.4 ± 5.9	0.006
Side of ectopic mass			0.277
Left	35(57.3)	27(47.3)	
Right	26(42.7)	30(52.7)	
Previous caesarean			0.157
1	18 (29.5)	9 (15.8)	
2	4 (6.6)	8 (14)	
3	/	1 (1.8)	
Previous L/S	5 (8.2)	5 (8.8)	0.585
Previous myomectomy	1 (1.6)	/	0.871
Previous appendectomy	6 (9.8)	8 (12.3)	0.186
Pregnancy type			
Spontaneus	55 (90.2)	51 (89.5)	0.901
ART	6 (9.8)	6 (10.5)	
			0.89

**BhCG:** Beta human chorionic gonadotropin, **USG:** Ultrasonography, **L/S:** Laparoscopy, **ART:** Assisted reproduction technique

The operative and postoperative data of the groups are presented in Table-II. When the operative and postoperative data of the groups were examined, the operation time was found to be lower in the uterine manipulator group than in the group where uterine manipulators were not used (P < 0.001). No difference was noted between the groups in terms of estimated blood loss, haemoperitoneum, operative and postoperative complications and postoperative hospitalization. Return of bowel function was similar in both groups. There was no statistically significant difference between the groups in terms of postoperative hospital stay (P=0.945). In terms of intraoperative complications, cervical laceration that required the use of sutures was observed in one patient and uterine perforation was observed in another patient in the uterine manipulator group.

**Table-II:** Operative data and complications.

Operation time (min)	65 (45–130)	50 (25–118)	<0.001
Estimated blood loss (ml)	130 (90–280)	110 (70–240)	0,548
Haemoperitoneum	10 (16.4)	7 (12,5)	0.37
***Operation type***			
Salpengectomy	51 (83,6)	48 (84,2)	0.604
Salpingostomy	10 (16,4)	9 (15,8)
***Complications***			
Cervical laceration	1 (1,6)	/	
Subcutaneous emphysema	7 (11,5)	6 (10,5)	0.552
Uterine perforation	1(1,6)	/	
Intestinal injury	/	/	
Bladder injury	/	/	
Haemorrhages requiring transfusion	/	/	
Postoperative fever	2 (3,3)	2 (3,5)	0.665
Return of bowel function (h)	8 (4–24)	8 (5–12)	0.075
Urinary catheter time (h)	4 (3–7)	4 (3–6)	0.631
Duration of postoperative hospital stay (days)	1 (1–2)	1 (1–2)	0.945

## DISCUSSION

In this study, we aimed to investigate the effect of uterine manipulator use on the outcome of laparoscopy in patients with tubal ectopic pregnancy. To our knowledge, this is the first study to evaluate such outcomes in the literature.

The surgical treatment of ectopic pregnancy is indicated in patients who require a second dose of methotrexate treatment due to treatment failure, hemodynamically unstable patients, patients with rupture, hypotension, anaemia, gestational sac with a diameter of more than 40 mm on ultrasonography, patients who cannot comply with the monitoring protocol from the time of diagnosis due to reasons such as pain lasting more than 24 hours, patients with contraindications to methotrexate, patients who require second surgery in the same session such as tubal ligation and those in whom methotrexate therapy is indicated.^7^ In the surgical treatment of ectopic pregnancy, laparoscopy is an effective and safe option in which the operation time and duration of hospital stay are shorter and events of requiring a blood transfusion is less compared with that in laparotomy.^8^

Uterine manipulators are frequently preferred in laparoscopic hysterectomy because they reduce the incidence of ureteral injuries and facilitate colpotomy and are successful in maintaining pneumoperitoneum after colpotomy.^9^ In addition, uterine manipulators are used in diagnostic procedures that require the anteroposterior and lateral movements of the uterus, tubal ligation, uterine niche repair, treatment of caesarean scar pregnancy, treatment of ectopic pregnancy and laparoscopic excision of ovarian masses and uterine fibroids.^10^–^13^ As a result, uterine manipulators are frequently used in the laparoscopic treatment of ectopic pregnancy and in almost all operative or diagnostic gynaecological laparoscopic procedures because they are thought to reduce complications by facilitating dissection via contralateralisation and increasing the field of view of the surgeon.^14^,^15^

However, complications may occur during the insertion or use of uterine manipulators. More than half of the cervical injuries that occur during the insertion of uterine manipulators are caused by the tenaculum, which is frequently used to hold the cervix.^16^ In our study, cervical laceration was reported in one (1.6%) patient during uterine manipulator insertion and bleeding was controlled by suturing. In the literature, uterine perforation is reportedly associated with the use of RUMI manipulators for chromopertubation and when a large amount of fluid is mistakenly sent into the tip balloon.^17^ After uterine perforation with the Hohl manipulator, penetration into the uterus or adjacent organ, such as intestinal injury and uterine artery pseudoaneurysm due to manipulator, has also been reported. In our study, RUMI manipulators were used as the uterine manipulator, which resulted in perforation in the fundus of uterus in one patient, which is consistent with the literature.^6^,^18^ Furthermore, for manipulators with multiple parts, it should be kept in mind that manipulator parts can be retained in the vagina due to errors in counting these parts after the operation. Ellett et al. reported gas packs retained in the vagina that were placed due to cervical bleeding in two patients after the repair of vaginal injuries following the use of uterine manipulators, and this is important in terms of the medico-legal consequences of gas tampons not counted in vaginal use in clinical practice.^19^ Since there was no need for cervical cups in our study group, all RUMI uterine manipulators comprised two parts, the tip and the shaft, and laparoscopy was concluded after pelvic organs were re-examined for perforation after cervical and vaginal examination following laparoscopy and removal of manipulator.

In a study on the use of uterine manipulators in laparoscopic sterilisation involving 164 patients, Prasad et al. did not report any difference in terms of complications between the groups that were operated with or without uterine manipulators even in the presence of a previous history of abdominal surgery, which is a finding consistent with the results of our study.^20^ In our study group, there was no difference between the groups in terms of other abdominal surgery, including caesarean section and complications. In our study, the return of bowel function was found to be 8 hours on average for both groups, a period consistent with that reported in the literature, and no significant difference was found between the groups (P = 0.075).^21^ Although lower urinary tract injuries are often encountered in laparoscopic hysterectomy and laparoscopy assisted vaginal hysterectomy operations, they were not reported in our study groups, which is consistent with the findings of a review by Satitnirmai and Manonai in 2017.^22^,^23^

Although uncommon, bowel injuries in gynaecological laparoscopy could be lethal and are more frequently observed in laparoscopic sacrocolpopexy and laparoscopic hysterectomy performed due to benign or malignant indications, but they are less frequent in adnexal operations.^24^ In our study, bowel injuries were not observed in both the groups. When the studies comparing single-port and traditional methods in the laparoscopic treatment of ectopic pregnancy were examined, no significant difference was noted between the groups in terms of operative data and complications, and this method is reported to be a safe and effective method independent of the type of ectopic pregnancy and hemodynamic instability.^25^ Prospective studies on ectopic pregnancy surgery with single port without uterine manipulators will make further contributions to the literature.

### Limitation of our study

The main limitation of our study is that it had a retrospective design; however, we aimed to mitigate this limitation with patient selection criteria. Furthermore, our study being the first article in the literature on the efficacy of uterine manipulator in laparoscopic surgical treatment is one of the strengths of this study.

## CONCLUSION

In conclusion, the use of uterine manipulators in the laparoscopic treatment of ectopic pregnancy resulted in no statistically significant difference in terms of operative complications and postoperative data. Prospective randomised multicenter studies including a larger sample size are warranted to corroborate the findings of this study and will contribute to the literature.

### Author`s Contribution:

**EB** conceived, designed and did statistical analysis & editing of manuscript.

**AGK, AB, VE & SOT** did data collection and manuscript writing.

**AHI** did review and final approval of manuscript.
